# Pupillometry Assessment of Speech Recognition and Listening Experience in Adult Cochlear Implant Patients

**DOI:** 10.3389/fnins.2020.556675

**Published:** 2020-11-06

**Authors:** Francesca Yoshie Russo, Michel Hoen, Chadlia Karoui, Thomas Demarcy, Marine Ardoint, Maria-Pia Tuset, Daniele De Seta, Olivier Sterkers, Ghizlène Lahlou, Isabelle Mosnier

**Affiliations:** ^1^INSERM U1159 Réhabilitation Chirurgicale Mini-Invasive Robotisée De l’Audition, Paris, France; ^2^Assistance Publique Hôpitaux de Paris Sorbonne Université, Service Oto-Rhino-Laryngologie (ORL), Unité Fonctionnelle Implants Auditifs, Groupe Hospitalier Pitié-Salpêtrière, Paris, France; ^3^Department of Sense Organs, Faculty of Medicine and Dentistry, Sapienza University of Rome, Rome, Italy; ^4^Oticon Medical, Vallauris, France; ^5^INSERM U1120 Génétique et Physiologie de l’Audition, Paris, France; ^6^APHP Sorbonne Université, Service ORL, GH Pitié Salpêtrière, Paris, France

**Keywords:** cochlear implant, listening effort, pupillometery, pupil dilatation, speech in noise

## Abstract

**Objective:**

The aim of the present study was to investigate the pupillary response to word identification in cochlear implant (CI) patients. Authors hypothesized that when task difficulty (i.e., addition of background noise) increased, pupil dilation markers such as the peak dilation or the latency of the peak dilation would increase in CI users, as already observed in normal-hearing and hearing-impaired subjects.

**Methods:**

Pupillometric measures in 10 CI patients were combined to standard speech recognition scores used to evaluate CI outcomes, namely, speech audiometry in quiet and in noise at +10 dB signal-to-noise ratio (SNR). The main outcome measures of pupillometry were mean pupil dilation, maximal pupil dilation, dilation latency, and mean dilation during return to baseline or retention interval. Subjective hearing quality was evaluated by means of one self-reported fatigue questionnaire, and the Speech, Spatial, and Qualities (SSQ) of Hearing scale.

**Results:**

All pupil dilation data were transformed to percent change in event-related pupil dilation (ERPD, %). Analyses show that the peak amplitudes for both mean pupil dilation and maximal pupil dilation were higher during the speech-in-noise test. Mean peak dilation was measured at 3.47 ± 2.29% noise vs. 2.19 ± 2.46 in quiet and maximal peak value was detected at 9.17 ± 3.25% in noise vs. 8.72 ± 2.93% in quiet. Concerning the questionnaires, the mean pupil dilation during the retention interval was significantly correlated with the spatial subscale score of the SSQ Hearing scale [*r*(8) = −0.84, *p* = 0.0023], and with the global score [*r*(8) = −0.78, *p* = 0.0018].

**Conclusion:**

The analysis of pupillometric traces, obtained during speech audiometry in quiet and in noise in CI users, provided interesting information about the different processes engaged in this task. Pupillometric measures could be indicative of listening difficulty, phoneme intelligibility, and were correlated with general hearing experience as evaluated by the SSQ of Hearing scale. These preliminary results show that pupillometry constitutes a promising tool to improve objective quantification of CI performance in clinical settings.

## Introduction

For hearing-impaired people, listening and understanding speech in noisy situations is a difficult task, requiring substantial cognitive effort and often associated with increased self-reported fatigue (Alhanbali et al., 2017). In particular, patients with cochlear implants (CIs) often experience high levels of listening effort in everyday life listening situations due, among other causes, to the spectral reduction that CIs impose on incoming sounds (Winn et al., 2015). This constant subjective experience of effortful listening is correlated with degraded speech-in-noise performance and experienced hearing handicap (Alhanbali et al., 2018). As the severity of hearing loss increases, or as the environment becomes more challenging, hearing-impaired individuals are forced to engage more cognitive resources to direct attention and focus on fine-acoustic details of speech. This leads to increased mental load and concentration needed to identify, recognize, and understand auditory information, negatively affecting other cognitive functions such as memory abilities (Ng et al., 2013; Zekveld et al., 2013). This cognitive side effect of hearing loss can have negative consequences on an individual’s well-being and quality of life (Hornsby et al., 2016). Moreover, as highlighted in the Framework for Understanding Effortful Listening (FUEL) model, people who have to make constant cognitive efforts will not necessarily overcome their listening problem; on the contrary, they may develop chronic stress and failure-avoidance strategies, leading them to progressively disengage from social relationships. Having to make constant listening efforts is therefore likely to have a negative impact on health and cognition in general (Pichora-Fuller et al., 2015; Pichora-Fuller, 2016). The hypothesis of a chronic excessive cognitive load existing in hearing-impaired patients is indeed sustained by several recent researches, in which it has been demonstrated that even moderate levels of hearing loss constitute a risk factor for dementia and cognitive decline (Lin, 2011; Lin et al., 2013; Deal et al., 2017).

In this context, one limitation of conventional audiometry tests currently used to evaluate hearing loss or quantify the efficiency of a hearing treatment such as CIs is that they do not accurately reflect cognitive effort that patients experience when listening. In the clinic, auditory performance is routinely evaluated through psychoacoustic tests, often conducted in quiet only, and in order to reach equal performance, certain patients may need a much greater effort than others. Furthermore, these tests are conducted in laboratory conditions and cannot reflect the efforts patients make in their everyday lives and/or the cognitive fatigue they may experience. Identifying new objective measures of hearing abilities, which could capture the interindividual variability of cognitive load associated with individual listening experience, could help improve long-term follow-up of CI users and refine inclusion criteria for CI candidates.

To date, different options have been considered to measure cognitive effort related to listening in difficult conditions (see Peelle, 2018 for a recent overview). One approach consists in evaluating the impact of increased effort directly on behavioral outcomes, with the use of dual-task paradigms, or the measurement of response-time increases (Hughes and Galvin, 2013; Antoniou and Wong, 2015; Pals et al., 2015). Another approach consists in measuring physiological correlates of cognitive effort, directly from the central nervous system, using electrophysiological or neuroimaging methods (e.g., Weisz and Obleser, 2014; Marsella et al., 2017). A third possibility is to observe the indirect reflection of cognitive load on the autonomous nervous system and measure pupillary dilation (Zekveld et al., 2010; Koelewijn et al., 2012, 2014, 2015; Steel et al., 2015; Winn et al., 2015; Kramer et al., 2016; Wagner A. E. et al., 2016; Wang et al., 2016), which in recent years has become a popular technique in the field of listening effort evaluation.

In particular, the pupillary response was well documented as being “*a correlate of cognitive intensity, an indirect, not causally linked*” marker of cognitive load (Just and Carpenter, 1993). Averaging pupillary responses evoked by different mental or sensory processes allows obtaining task-evoked pupillary responses (TEPRs), like electroencephalographic signal analysis with event-related potentials (ERPs). Metrics used to characterize the TEPR are the mean dilation, the maximal value, and the latency of the peak response to a particular stimulation, the allocation of more resources (higher load) leading to larger pupil dilation. It is, however, a complex experimental model with several crucial variables, as previous works have shown that TEPR characteristics are modulated by the complexity of the cognitive task, by attentional or memory load, and by motivation (Kahneman and Beatty, 1966; Kahneman, 1973; Ben-Nun, 1986; Peelle, 2018; Zhao et al., 2019; and see Beatty, 1982; Beatty and Lucero-Wagoner, 2000; Winn et al., 2018; Zekveld et al., 2018 for reviews).

When studying speech processing in hearing-impaired subjects, pupil dilation showed to be an indirect reflection of intelligibility. Kramer et al. (1997) studied mean pupil dilation in normal-hearing and hearing-impaired people listening to sentences presented at different signal-to-noise ratios (SNRs). Their results showed that mean pupil dilation was proportional to the difficulty of the task, being larger when the SNR was lower and being in general larger in hearing-impaired than in normal-hearing participants. These results suggested that pupillometry could be used as a proxy of listening difficulty in a hearing-impaired population. Zekveld et al. (2010) extended these observations by measuring peak dilation, latency of the peak, and mean dilation of the TEPR associated with listening to sentences at different SNRs, corresponding to individually adjusted intelligibility levels. These authors reported that peak dilation amplitude, peak latency, and mean pupil dilation increased with increasing noise level, suggesting a modulation by listening effort, which would be more important when the noise level becomes higher. These authors also reported that pupil dilation was larger for missed trials (i.e., incorrectly repeated sentences) than for hits (i.e., correctly repeated sentences), suggesting a potential direct link between listening performance and pupil dilation measures. Others have since shown that pupillometry can be used to assess listening effort during speech-in-speech perception in the presence of speech maskers (Koelewijn et al., 2012, 2014). Furthermore, Kuchinsky et al. (2013) demonstrated that pupil dilation was larger and more delayed at lower SNR and in the presence of a lexical competitor, thereby confirming that pupillometry provides an additional dimension to evaluate word perception and comprehension than behavioral measures alone. If pupillometry can be used as a measure of task complexity and processing load in normal-hearing and hearing-impaired populations during speech perception, it may be used to obtain further information regarding the processing load engaged during speech processing by CI users, which could be highly relevant in terms of clinical applications. First studies in this field showed that normal-hearing participants who were presented with acoustic stimuli that were processed to mimic CI stimulation showed increased listening effort (Pals et al., 2013), and a pupillary response that was proportional to the amount of spectral reduction imposed to the stimulation (Winn et al., 2015). To our knowledge as of today, only three papers reported pupil dilation responses in CI users. Winn et al. (2016) and Winn and Moore (2018) explored the ability of CI users to benefit from contextual information during sentence comprehension and measured their pupil dilation during this task. They first observed that CI users showed in general higher effort levels than normal-hearing controls performing the same task, as evidenced by larger baseline-corrected mean dilation values, but also that CI users benefited less and with a delayed effect from contextual cues than normal-hearing participants. They further showed that this later-occurring, and longer-lasting “repair” process observed in CI users, could easily be perturbed, and even canceled by later-occurring sounds, especially when these were intelligible utterances (Winn and Moore, 2018). More recently, Wagner et al. (2019) reported that CI users showed increased variability in their evoked pupil responses compared to matched normal-hearing participants, suggesting that this variability could be clinically meaningful and could be an index of processing load in individual patients. Even if these first studies highlight the interest of pupillometry to explore cognitive processing involved during listening tasks in CI users, the potential of pupillary responses as a clinical objective tool to evaluate CI performance remains to be explored.

The aim of the present study is to study the pupil dilation in response to word identification in CI users, and to understand whether this approach can constitute a promising complement to psychoacoustics tests performed in the clinic. We hypothesized that when task complexity (addition of background noise) increases, pupil dilation markers such as peak dilation and the latency of the peak dilation will increase in CI users as already observed in normal-hearing and hearing-impaired users (Kramer et al., 1997; Zekveld et al., 2010; Kuchinsky et al., 2013). A second objective will be to assess the relationship between performance during speech recognition and multiple components of pupil response, in order to establish if some of them can be used as objective measures of speech perception performance in CI users. We hypothesized that pupil dilation would be larger in incorrect than in correct trials as suggested by Zekveld et al. (2010) for normal-hearing subjects. A further objective is to investigate the presence of a correlation between the objective pupillary response and the subjective effort perceived by the patient, or the quality of listening in daily life.

To do so, we ran an experiment in which we combined pupillometry to speech audiometry measures performed in experienced CI users and the evaluation of subjective hearing quality in the same patients.

## Materials and Methods

### Participants

Volunteers enrolled in this prospective interventional study were adult patients who underwent CI surgery between 1998 and 2016 in a tertiary referral implant center. The consistent criterion for CI candidacy was the presence of a bilateral postlingual severe-to-profound hearing loss with speech recognition scores ≤ 50% for open-set disyllabic words presented at 60 dB sound pressure level (SPL) in quiet, in the best aided condition, after verification of the optimal fitting of a hearing aid (Guidelines from the “Haute Autorité de Santé”, January 2012^1^). Inclusion criteria were native adult French speakers or patients fluent in French who were implanted with an Oticon Medical CI system (Oticon Medical, Vallauris, France). Patients should have used their CI for at least 1 year or more and have word recognition scores greater than or equal to 10% for disyllabic words presented in quiet, in the best aided condition. They should have normal or corrected-to-normal vision. Because cognitive abilities can influence cognitive load and pupil dilation measures (Zekveld et al., 2011), we also evaluated our participants’ cognitive functions with the Montreal Cognitive Assessment (MOCA) neuropsychological test, presented in its visual version. The MOCA test has already been used in the literature to screen for cognitive impairments (Pendlebury et al., 2015), and it has been validated for CI users (Ambert-Dahan et al., 2017; Lin et al., 2017). The MOCA test was administered in its complete form, on paper during the inclusion visit. A score out of 30 points was collected. A score above 26 was considered as normal. The present study was approved by the ethics committee: *Comité de Protection des Personnes*, Ile de France IV, Paris (ID-RCB N°2017-A00318-45). Written informed consent was obtained from each patient before their enrolment in the study. The present study followed the ethical principles of the World Medical Association Declaration of Helsinki. Written, informed consent was obtained from all included subjects for the publication of any potentially identifiable images or data included in this article. The protocol was registered in the U.S. National Library of Medicine ClinicalTrials.gov database (identifier NCT03212924).

### Stimuli and Conditions

Isolated monosyllabic words were extracted from a CD recording (*listes cochléaires de Lafon*, Collège national d’audioprothèse, Paris, France) and stored as. wav files (stereo, 44.1 kHz, 16 bits). Each word was embedded into a new 10 s. wav file using a Matlab© script (MathWorks, Natick, MA, United States). Two sets of audio files were generated corresponding to the two experimental conditions: for the Quiet condition, only the target-word was present, with an onset at 5 s. For the Noise condition, a speech-shaped noise extract was added to the target-word, at an SNR of +10 dB in a S0N0 configuration (i.e., where the target-words and the speech-shaped noise are presented from the same speaker in front of the subject), starting at 2 s and stopping at 8 s, the target-word starting at 5 s. All audio files were normalized in global intensity to avoid changes in global amplitude of the stimuli due to the mixing procedure (i.e., rms normalization). These audio files were then embedded in.mov video files, generating two sets of stimuli to be displayed in an eye-tracking system allowing continuous pupil-size measure. The initial list structure of the Lafon test (20 lists of 17 words) was preserved, leading to a total set of 680 available stimuli, 340 per condition.

#### Trial Structure

**FIGURE 1:**
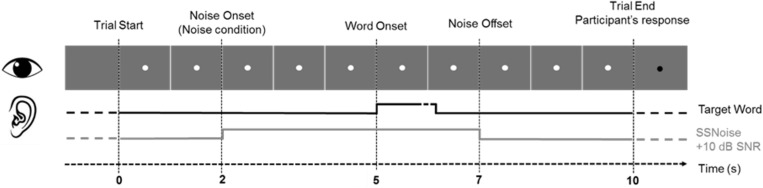
General trial structure and timing of events.

Each trial lasted a total of 10 s (Figure 1) and started with the display of a central fixation point, followed by a 2 s wait to ensure pupil size stabilization. At 2 s, the noise started in Noise conditions. Target-words were always presented at 5 s after trial onset in both conditions. In noise trials, the noise was faded-out at 8 s, the trial went on until 10 s, when the fixation point changed color. Patients were instructed to repeat what they had heard at the end of the trial. Responses were noted down by the experimenter and the next trial began. Pupil dilation was measured in continuous during the 10 s of each trial.

Before the start of the experiment, each participant was presented with one training list, in order to familiarize with the general experimental procedure and timing of events. Participants were then presented with six 17-words lists, three per condition (Quiet or Noise), representing 51 trials per condition per participant. Participants started either by the Quiet or the Noise condition, lists, and conditions were randomized and balanced across participants ensuring that none of the target-words were repeated between conditions. Short breaks of about 5 min were observed between lists, the total duration of the session including breaks was kept below 45 min to avoid excessive fatigue.

### Procedure

Tests were performed in a sound-treated room in a free-field configuration, using two loudspeakers positioned at 1 m distance in front of the patient. Patients were tested in their best-aided condition. Stimuli were presented at 65 dB SPL. Pupil diameter data were gathered for both eyes using a Tobii^®^ TX300 eye-tracking system (Tobii Technology AB, Danderyd, Sweden). This eye-tracker is composed of a 23” TFT monitor placed at forearm distance in front of the participant and an infra-red diodes sensor placed below the screen, monitoring gaze movements and pupil size at 300 Hz (3.33 ms samples). The system is non-obtrusive and non-invasive and shows a high tolerance to head movements; pupil dilation can thus be measured without chinrest, head-fixation, or the wearing of any extra-device. Before any measurement, a calibration phase was performed, to ensure that the positioning of the participant was optimal. Illumination inside the sound booth was kept constant during the entire experiment (no natural light, artificial light constant) and at the same level for all participants.

#### Questionnaire-Based Evaluation of Subjective Listening Effort and Subjective Hearing Abilities

Besides the audiometry/pupillometry session, patients were tested for subjective listening effort and subjective hearing abilities.

Two visual analog scales (VAS), requiring participants to indicate their subjective listening effort in quiet and in noise by placing a mark on a 10 cm long line graded from 0 (no effort) to 10 (maximal effort), were used. The VAS scoring was calculated as the measure in cm from the 0 value to the mark placed by the patient. This subjective test was directly associated to the pupillometry measures as patients were asked to rate their subjective effort level after completion of each condition (Quiet or Noise), during the pupillometry session.

Subjective hearing abilities were evaluated using the Speech, Spatial, and Qualities (SSQ) of Hearing Scale (Gatehouse and Noble, 2004), using the validated French version (Moulin et al., 2015).

The questionnaire includes three subscales, evaluating speech comprehension, spatial hearing, and hearing quality, with a total of 49 items scored on a scale from 0 (total inability) to 10 (full capacity). Higher scores indicate higher subjective hearing abilities. The SSQ questionnaire was filled independently of the pupillometry measures, during the inclusion visit.

#### Pupillometry Data Processing

Continuous pupil diameter values (mm) were extracted from raw data for each trial. From the original 10 s trials, the first and last seconds were discarded, generating 8 s recordings for pre-processing and analysis, performed using *ad hoc* scripts developed under SciPy a Python-based library^2^. Data were first averaged over the two eyes, except if one of the two eyes was of excessive low quality (i.e., more than 20% sample rejection), then data from only one eye were considered. For each individual trial, data showing a difference of more than three standard deviations of the mean (SD) below the average trace were coded as eye-blinks or absent data due to head-movements. Trials for which eye-blinks exceeded 20% were rejected from further analyses. In the remaining trials, eye-blinks and missing data were replaced by linear interpolation taking the five samples before the gap and the eight samples after the gap into account. Finally, a five-point moving average smoothing filter was applied in order to remove high-frequency artifacts.

Percent change in event-related pupil dilation (ERPD) were then calculated, per each individual trial and participant, according to the formula described in Wagner et al. (2019), % change ERPD = 100x (observation – baseline)/baseline. Baseline was defined as the one-second period preceding the onset of the noise (i.e., time 0 in all graphical representations). Grand averages were generated per participant and conditions by averaging baseline-corrected % changes in ERPDs together. Mean pupil dilation was extracted in three different time-windows. “Background” was defined as the mean value over the 0.5–3.5 s time-window, corresponding to the time between the pupil response to the noise, occurring 0.5 s after noise onset in the Noise condition, and the pupil reaction to the presentation of the target-word, occurring 0.5 s after target-word onset. The “Peak” time-window lasted from 3.5 to 5.5 s and corresponded to the phasic dilation response evoked by the presentation of the target-word. The retention interval was defined from 5.5 to 7 s and corresponded to the progressive return to baseline following the peak dilation to the presentation of the target-word. Finally, the latency of the peak corresponding to the time of occurrence of the maximal dilation value in the peak time-window was also extracted.

### Statistical Analyses

Analyses were conducted in RStudio Team (RStudio Inc., Boston, MA, United States), in Statistica Version 10 (StatSoft France, Maisons-Alfort, France) and GraphPad Prism 7.04 (GraphPad Software, Inc., La Jolla, CA, United States). For each analysis, linear mixed-effect (LME) models with a random intercept for participants were fitted to the data (lmerTest package; Kuznetsova et al., 2017). To further explore the data, repeated-measures ANOVA and *t*-test were conducted in. For all analyses, statistical significance was defined as *p* ≤ 0.05. No missing data were reported. Further correlational analyses were run to identify correlations between pupillometry markers and behavioral results from the speech audiometry and the different questionnaires and subjective measures.

## Results

Twelve patients were originally included in the study, one of them had to be excluded from further analyses because of the poor quality of its pupillometry measure (more than 20% + missing data or eye-blinks), and one more patient could only perform two lists of words per conditions. In order to keep the balance of number of trials performed across participants, which could potentially influence global fatigue level and thus pupil dilation measures, we decided to also exclude this dataset from further analyses. Ten participants therefore provided data of sufficient quality to be included in further analyses (*N* = 10). Patients’ demographics and cochlear implantation data are provided in Table 1. Included patients were all users of Digisonic SP (Oticon Medical, Vallauris, France) CI systems (two unilateral CI users, four bilateral CI, three bimodal CI/Hearing aid, and one Binaural device, see Bonnard et al., 2013). Patients were on average aged 63 ± 9 years (range: 51–79 years), and were all experienced CI users with an average CI experience duration of 8 ± 3 years (range: 4–13 years). Results from the MOCA evaluation led to an average total score of 26.6 ± 3.7 (min = 17, max = 30) on a 30-point scale and the sub-test word-recall to an average of 4.1 ± 0.7 (min = 3, max = 5) on a five-point scale. On average, patients included in the study had normal MOCA and word-recall scores, except one patient with a total score of 17. However, pupil dilation data could be acquired in this patient as well, and he/she could perform all requested tasks. The MOCA scores did not correlate with any of the other measures performed in the present study, neither the speech audiometry scores nor the pupil dilation data.

**TABLE 1:** Patients’ demographics.

Patient Id	Gender	Age (years)	Etiology	Best-aided condition	CI experience (years)	Cochlear implant type	MOCA Total Score
							
01	F	68	Unknown	Bimodal CI/HA	5.8	Digisonic SP	25
02	M	51	Familial	Binaural	8.4	Digisonic Binaural	27
03	M	59	Progressive	Unilateral CI	10.6	Digisonic SP	27
04	F	60	Otosclerosis	Bilateral CI	8.3	Digisonic SP	28
05	F	54	Unknown	Unilateral CI	10.6	Digisonic SP	30
06	M	69	Meningitis	Bilateral CI	4.7	Digisonic SP	26
07	M	61	Otosclerosis	Bilateral CI	6.4	Digisonic SP	29
08	F	56	Ototoxicity	Bilateral CI	11.6	Digisonic SP	27
09	F	79	Otosclerosis	Bimodal CI/HA	3.8	Digisonic SP	17
10	M	70	Genetic	Bimodal CI/HA	12.6	Digisonic SP	30
**Average or count**	5 f/5 m	63	–	–	8.3	–	26.6
**SD**	–	9	–	–	3.0	–	3.7

*Patient Id: patient index, Gender: F: female, M: male, Best-aided condition: Bimodal: CI in one ear and hearing aid (HA) in the other, Binaural: Digisonic Binaural^®^ device. MOCA evaluation total score, max = 30 points.*

### Speech Audiometry

**FIGURE 2:**
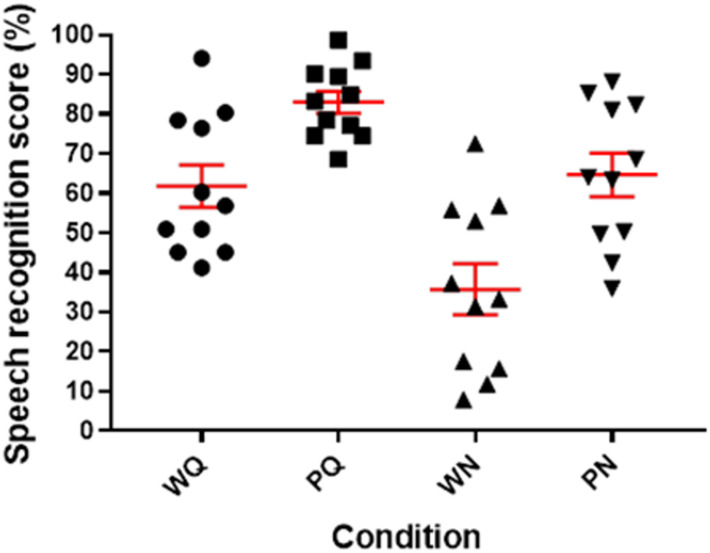
Scatter-plot of individual speech audiometry scores obtained in quiet (Q) and in noise (N) percent correct identification scores for Lafon words (W) and phonemes (P), in red: mean and standard error of mean.

Figure 2 shows the individual speech recognition scores obtained during the pupillometry measures. Recognition scores for words and phonemes were higher in Quiet, respectively, 60 ± 17.9 and 82 ± 9.5% than in Noise: 34 ± 21.5 and 63 ± 17.8%. Linear mixed model was applied to the data with random intercept for subject and defined fixed effect for test: [word (W) and phoneme (P)] and condition [Quiet (Q) and Noise (N)]. The analysis revealed an overall significant effect for both condition (*F*-value = 108.86, *p* < 0.001) and test (*F*-value = 138.76, *p* < 0.001) with the scores in Quiet being higher than in Noise (*t*-value = 10.43, *p* < 0.001) and scores for words recognition being lower than for phonemes recognition (*t*-value = −11.78, *p* < 0.001). *Post hoc* pairwise analysis for words recognition revealed that scores in Quiet were significantly higher than in Noise (*t*-ratio = −10.43, *p* < 0.0001). Similarly, phonemes recognition for Quiet was significantly higher than in Noise (*t*-ratio = −15.07, *p* < 0.0001).

### Pupillometry

#### Effect of Noise Onset on Pupil Dilation

**FIGURE 3:**
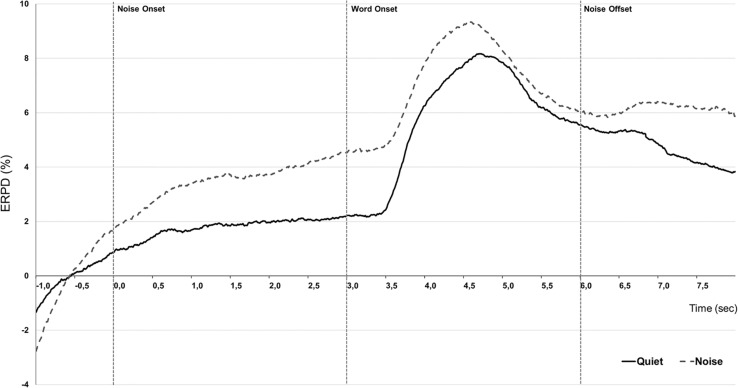
Effect of conditions. Grand-averaged (*N* = 10) pupillometry traces showing percent change in event-related pupil dilation (ERPD) for correctly recognized words, presented in Quiet (black line) or +10 dB speech-weighed noise background (gray dashed). Time is given relative to the onset of the noise in noisy background set at 0 s, words are presented at 3 s, and noise fades-out at 6 s.

Results from the pupil dilation measures obtained during speech audiometry were first analyzed taking only correctly recognized words into account. Visual inspection of the grand-averaged, baseline corrected curves (Figure 3) shows that pupil dilation was globally larger in the Noise condition, during three periods of the recording that we defined as three different time-windows for further analyses. A first rise in pupil dilation appears in response to the onset of the noise relative to the quiet period. This increase starts at 0.5 s after the onset of the noise and lasts until 3.5 s, this defines a first time-window for analysis: Background (0.5–3.5 s). A TEPR follows, occurring between 3.5 and 5.5 s relative to the onset of the noise, or approximately 0.5 s after the presentation of the target-word, defining a second time-window for analysis: Peak (3.5–5.5 s). Finally, traces tend to go back toward baseline values, until the end of the trials at 7 s after noise onset, in a thirds time-window: Retention interval (5.5–7 s). We first extracted mean pupil dilation values from the three defined time-windows for each patient (see Table 2 for average values of the different pupillometry markers analyzed).

**TABLE 2:** Detailed pupil dilation characteristics (correct trials).

	Time-windows (Percent change, %)			Peak characteristics	
Condition	Background (0.5–3.5 s)	Peak (3.5–5.5 s)	Relaxation (5.5–7.0 s)	Peak max (%).	Peak latency (from target-word onset, s)
**Quiet**					
Average	2.19	7.56	5.04	8.72	1.65
SD	2.46	1.20	1.66	2.93	0.15
**Noise**					
Average	3.47	8.17	6.58	9.17	1.57
SD	2.29	3.75	3.05	3.25	0.19

*Percent change in event-related pupil dilation (ERPD) averaged over the three time-windows, for the condition Quiet (upper rows) and Noise (lower rows). Peak characteristics: Peak max.: maximal percent change in event-related pupil dilation (ERPD) during peak and latency of peak expressed in seconds following the onset of the target-word.*

#### The Evolution of Mean Pupil Dilation by Time-Window and Word Recognition Condition

**FIGURE 4:**
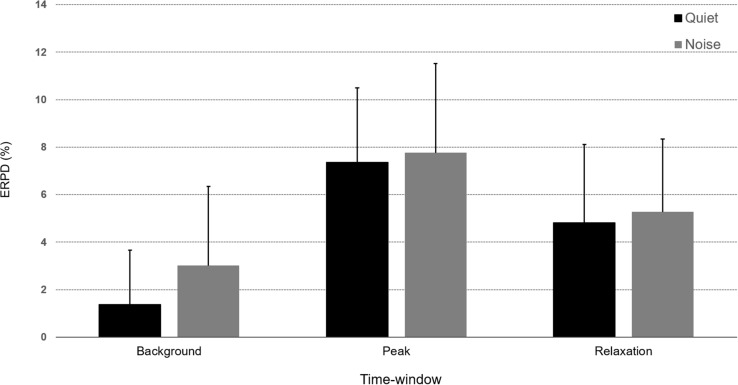
Bar-graphs representing percent change in event-related pupil dilation (ERPD) averaged over the three time-windows (Background, Peak, and Retention interval) for correct words presented in the Quiet (black) or Noise (gray) conditions. Error bars represent standard deviation of the mean.

In order to assess the overall course of mean pupil dilation in regards to time evolution in word recognition performance (Figure 4), an LME analysis was conducted with defined fixed effects of Condition (Quiet, Noise), Time-window (3: Background, Peak, Retention interval) and a random intercept for patients. The model describes a main effect of Condition (*F*-value = 32.45, *p* < 0.0001) and a main effect of Time-window (*F*-value = 212.65, *p* < 0.0001) but no significant interaction (*F*-value = 1.87, *p* = 0.15). In Quiet condition, *post hoc* pairwise analyses revealed that mean dilation was higher at peak window in comparison to background and retention intervals (respectively, *t*-ratio = −15.45, *p* < 0.0001 and *t*-ratio = 7.23, *p* < 0.0001) as well as significantly higher mean dilation in background interval than in retention interval (*t*-ratio = −8.21, *p* < 0.0001). The presence of noise interfering with word perception was associated to increased mean pupil dilation in CI users with significant effects starting during the background interval (Q vs. N, *t*-ratio = −3.68, *p* = 0.005) maintained during the peak response but not significant (Q vs. N, *t*-ratio = −1.67, *p* = 0.49) and until the retention interval with a significant effect (Q vs. N, *t*-ratio = −4.42, *p* = 0.0003).

To determine if pupil dilation measures were correlated with speech perception, we ran a first correlation analysis on speech audiometry scores and mean pupil dilation during the three identified time-windows. No significant correlation was observed between mean pupil dilation values extracted from the three time-windows and any of the speech perception scores.

#### Characterization of Peak Dilation Max Value and Latency by Speech Performance Condition

An analysis performed on the peak characteristics, including max peak value and peak latency, revealed a significant increase of maximum peak value, rising from an average 4.99% ERPD (SD = 2.45) in the Quiet condition, to 6.58% ERPD (SD = 3.29) in the Noise condition: paired *t*-test: *t*(9) = −4.34, *p* = 0.0004. This effect was on the contrary not significant on peak latency, measured at an average 1.65 s (SD = 0.15) in Quiet and 1.57 s (SD = 0.19) in Noise, *t*(9) = −1.35, *p* = 0.183. The effect observed on the mean pupil dilation during the peak time-window was confirmed on the max peak value, the Noise condition being associated with larger peak values. The latency of the peak was not significantly influenced by the presence of noise.

The correlational analysis revealed significant correlation coefficients between the peak latency observed in noise and speech recognition scores. The latency of the peak pupil dilation measured in the Noise condition was significantly correlated to: Words in Quiet, *r* = −0.84, *p* = 0.0023; Word in noise score, *r* = −0.68, *p* = 0.0314; Phonemes in Quiet *r* = −0.87, *p* = 0.0001; and Phonemes in Noise *r* = −0.75, *p* = 0.0117. This observation suggests that the pupil peak latency measured in noise could be a generic proxy of speech perception performance in CI users.

#### Effect of Successful Speech Performance on Pupil Dilation

**FIGURE 5:**
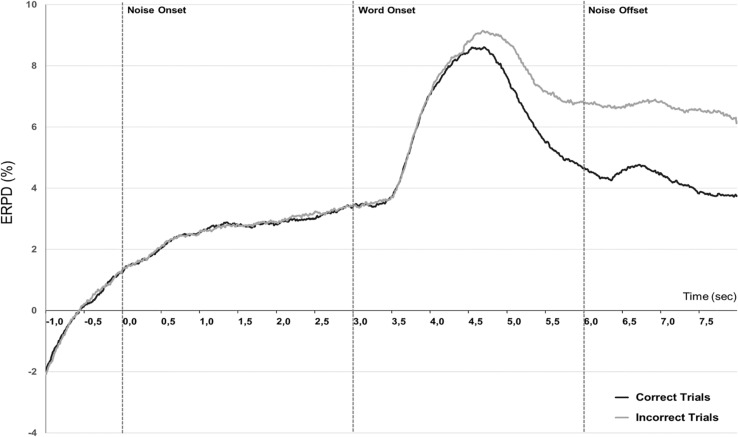
Performance effect. Grand-averaged (*N* = 10) pupillometry traces showing percent change in event-related pupil dilation (ERPD) for correctly recognized words (black line) or incorrect trials (gray line), independently of the listening condition.

In order to determine how these measures were influenced by task performance, we averaged together the trials leading to correct word identification and trials leading to incorrect or no response. The grand-averaged pupil dilation traces corresponding to correct and incorrect trials (Figure 5) show a potential modulation of pupil dilation by performance mostly in both the peak window and retention interval. In particular, it seems that the peak value, latency, and mean pupil dilation during the retention interval could be modulated by performance.

We extracted mean pupil dilation from individual averaged data over the same time-windows as preceding: Background (0.5–3.5 s), Peak (3.5–5.5 s), and Retention interval (5.5–7 s).

Linear mixed-effect model was fitted to the data considering individual mean pupil dilation as dependent variable and including factors Condition (2: Quiet, Noise), Performance (2: Correct, Incorrect), and Time-window (3: Background, Peak, Retention interval). The analysis revealed a significant main effect of the Time-window of analysis [*F*(2,99) = 89.76, *p* < 0.0001], a significant effect of Condition [*F*(2,99) = 11.46, *p* = 0.0010], a significant effect of Performance [*F*(2,99) = 11.05, *p* = 0.0012] as well as a significant interaction between Time-window and Performance [*F*(2,99) = 5.32, *p* = 0.006]. *Post hoc* comparisons corrected for multiple comparisons (HSD-Tukey) on this interaction showed a significant difference for the Quiet and Noise conditions only during the retention interval (*t-*ratio = 2.49, *p* = 0.017), during the return to baseline following the peak response.

#### Characterization of Peak Dilation Max Value and Latency by Successful Speech Performance

An analysis performed on the peak characteristics, including max peak value and peak latency, revealed no significant effect of performance on these two measures, maximal peak dilation was on average 9.79% ERPD (SD = 3.57) for correct trials, while it was of 10.47% ERPD (SD = 3.85) for incorrect trials *t*(9) = −0.96, *p* = 0.362. Peak delay was measured at an average of 1.54 s (SD = 0.21) after the onset of the target-word for correct trials and at an average of 1.71 s (SD = 0.23) after incorrect trials, a non-significant difference *t*(9) = −2.38, *p* = 0.21.

#### The Evolution of Mean Pupil Dilation by Time-Window and Phoneme Recognition Condition

**FIGURE 6:**
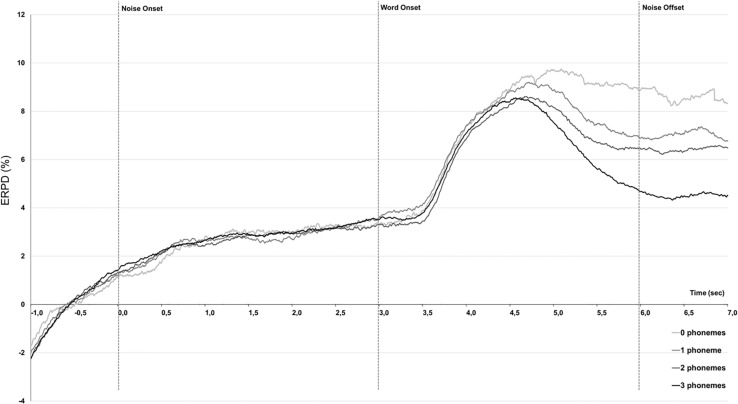
Grand-averaged (*N* = 10) pupillometry traces showing percent change in event-related pupil dilation (ERPD) analyzed across trials leading to 0, 1, 2, or 3 phonemes recognition.

Given that performance scored in correct words recognized had an impact on mean pupil dilation during the retention interval, we decided to run a complementary analysis on the pupillometry data. We averaged together all trials leading, respectively, to 0, 1, 2, or 3 correctly identified phonemes (Figure 6). Average pupil dilation values in the Background, Peak times windows, and retention interval were extracted and introduced as dependent variable in a mixed-model analysis. Participants, performance, and trials were treated as random effects in this analysis and Condition (2: Quiet, Noise), Phonemes (4: 0, 1, 2, or 3), and Time-window (3: Background, Peak, and Retention interval) as fixed factors.

The model found a significant effect of Phonemes (*F*-value = 10.48, *p* < 0.0001) and a significant effect of Time-window (*F*-value = 201.17, *p* = 0.0012). All other main effects and interactions remained non-significant as in particular, the Phonemes ^∗^ Time-window interaction was not further modulated by Condition (*F*-value = 1.78, *p* = 0.26). Besides, *post hoc* pairwise analyses revealed that through all three time-intervals, there were significant differences with the case 3 phonemes vs. 0- and 1- phonemes (*df* = 2807, *t*-ratio = 3.68, *p* < 0.0001; *df* = 2806, *t*-ratio = 3.42, *p* = 0.02, respectively) as long as with the case 2 phonemes vs. 0 phonemes (*df* = 2803, *t*-ratio = 5.22, *p* = 0.0023).

#### Subjective Hearing Quality Outcomes and Correlations With Pupil Dilation Data

##### SSQ questionnaire

Results from the SSQ questionnaires, were 4.0 ± 2.1 for the Speech category, 3.1 ± 2.5 for the Spatial subset, 5.7 ± 1.2 for the Qualities, and finally 4.4 ± 1.8 for the total SSQ score in our group of CI patients. No significant correlation was observed between the subjective SSQ evaluation and the speech recognition scores. A correlational analysis between the mean pupil dilation values observed in the three analyzed time-windows and SSQ scores, however, revealed many significant correlations at an alpha value of 0.05. After applying a Bonferroni correction (alpha = 0.002), two correlations remained significant and concerned the mean pupil dilation during the retention interval, that was significantly correlated with the Spatial subscale score *r*(8) = −0.84, *p* = 0.0023and with the Total SSQ score *r*(8) = −0.78, *p* = 0.0018. The significant correlation between the subjective hearing experience captured by the total SSQ score and the mean pupil dilation during the retention interval suggests an influence of the general hearing experience with the CI and the confidence of patients regarding their speech perception performance.

##### Subjective evaluation of listening effort

Results from the subjective evaluation of listening effort show that scores were significantly higher in the Noise condition (M = 7.3, SD = 2.2), than in Quiet (M = 4.0, SD = 2.0), paired *t*-test: *t*(9) = −4.03, *p* = 0.003, showing that participants were sensitive to the increased difficulty in speech recognition when noise was present. Correlations between subjective listening effort and speech recognition scores (eight correlations, Bonferroni-corrected alpha = 0.006) showed a significant correlation between correct word identification in Noise and VAS scores in noise *r*(8) = −0.88, *p* = 0.001. All other correlations remained non-significant and there was no significant correlation between subjective listening effort scores and mean pupil dilation in the three analyzed time-windows at Bonferroni-corrected *p*-levels.

## Discussion

With the present study, we explored the potential of pupillometry as an objective measure of speech perception in difficult hearing situations for CI users. We measured pupil dilation in 10 experienced CI users with various speech performance levels, placed in a clinically relevant situation corresponding to the usual setting used for speech audiometry testing open-set monosyllabic word recognition in quiet and in noise (+10 dB SNR) conditions.

We could identify three main time-windows during which our experimental conditions modulated pupil dilation values: a background period preceding the onset of the target-word, a peak dilation in reaction to the presentation of the target-word, and a retention interval, during which traces tend to return toward lower, baseline values in certain conditions only.

### Pupillometric Behavior in Speech Recognition Task in Case of Hearing Impairment

We could first of all observe that in all conditions the response of the pupillary dilation has drawn an inversed U-shaped curve, consistently with what is indicated in the FUEL and in other relevant articles in this field (Pichora-Fuller et al., 2016; Zekveld et al., 2018). These curves could be reflecting neural processing of listening effort in case of degraded speech presentation (i.e., especially through a CI). In fact, as discussed in the review by Peelle (2018), the inverse U-shaped curves were also observed in high-processing regions (e.g., of frontal cortex necessary for to extract meaning in degraded speech; Davis and Johnsrude, 2007). Second, the onset of the noise caused a first increase in pupil dilation compared to the quiet situation during the background, pre-target window. This increase was statistically significant at an average of 3.47 ± 2.29% in noise background, relative to the quiet control condition 2.19 ± 2.49%. These differences may highlight larger U-shaped curves from the beginning of the noise onset that would imply more listening effort in order to succeed in the task. Mean pupil dilation then remained with a larger tendency for the Noise condition during the Peak phase and the retention interval. The effect observed on the peak pupil dilation reproduces former works who also reported an increase of pupil size during speech perception, in normal-hearing and hearing-impaired participants. In CI users, pupil dilation measures are still quite rare, although the method offers several advantages compared to other objective measures in this particular population, such as the fact that it is completely non-invasive and very robust to electrical and magnetic artifacts (see, however, Winn, 2016 or Winn and Moore, 2018 for data in CI patients, and Winn et al., 2015 or Wagner A. et al., 2016, for CI simulation data). Studies implicating normal-hearing and hearing-impaired participants were conducted by Kramer et al. (1997), who reported an elevation of peak pupil dilation evoked by the presentation of sentences in noise at various SNR, showing that dilation was proportional to the difficulty of the listening situation (the lower the SNR the larger the dilation). In the same paper, authors reported that patients with hearing loss showed the same response profile but shifted toward higher dilation values and showing less decrease with easier listening conditions, suggesting that pupil dilation could reflect hearing handicap during speech perception. Zekveld et al. (2010) confirmed and extended these results. These authors showed in normal hearing participants that the peak dilation amplitude, peak latency, and mean pupil dilation increased with decreasing intelligibility. In our study, a first increase in pupil dilation is seen with the onset of background noise before the word presentation, this means that there is an increase in pupil dilation due to the perception of background noise only, which can be interpreted as an increase in concentration and/or motivation in the more difficult condition (Koelewijn et al., 2018) and thus of listening effort. One would argue that effort is an engaging task that would require motivation and this engagement would be fruitful especially if it is in the beginning and not sustained on a long term. During the presentation of the word, the mean dilation and maximal dilation were significantly increased by the more difficult listening situation created by the presence of noise. The effect on the latency was not observed but remained non-significant in our patients’ group (1.65 s in Quiet to 1.57 s in Noise). The absence of effect on peak latency could be explained by the relatively small size of our group (*N* = 10), compared to the ones reported in other works, or to the age of our participants. In their work, Zekveld et al. (2011) observed a correlation between the latency of the peak pupil dilation and age, that was stronger in the hearing-impaired group in which participants were also older (61 years on average). This is very similar to the mean age in our experimental group (62.6 years), and one could assume that in middle-aged patients, peak latency is increased and thus less sensitive to task-related parameters. Kuchinsky et al. (2013) observed the same trends in a group of 21 healthy normal hearing adults aged 73.1 years on average processing single words. However, the absence of a significant effect in our study may also reflect a more specific aspect of speech recognition in CI users, such as decision processes occurring after the presentation of the target-word, or the achievement of a plateau in the level of effort expended by these patients to understand speech, who are used to having to focus both in silence and in noise for all the duration of the clinical tests. In this context, it is worthy of consideration the strong motivation of CI patients in achieving success in clinical tests, since, as demonstrated by Koelewijn et al. (2018), when motivated people seem to be more persevering. Another hypothesis is that this absence of a significant effect on the peak delay was related to our experimental procedure requesting patients to remember the word they had heard and wait until the visual fixation point changed color before repeating what they had heard. This paradigm could impose less temporal stress on the response part of the task and leave more time for post-stimuli processes to develop and take place, decreasing the sensitivity of the peak latency as an index of stimulus processing. Pupil dilation was shown to be sensitive to working memory processes and an index of memory load during difficult speech perception.

This hypothesis was corroborated by the results of the analysis investigating the influence of performance on pupil dilation. We found that performance had no significant effect in our study on the peak dilation characteristics: mean dilation during the Peak time-window, max peak value, and peak delay, all non-significant. Besides, performance had a very clear influence on the retention interval just following the stimulus, similar to the trend reported by Winn et al. (2015). During this later time-window, correctly recognized words were associated with a faster return to baseline than incorrect trials.

### Phoneme Recognition and Pupillometric Assessment

Furthermore, a by-item analysis of averaged traces depending on the number of phonemes could show that the number of correctly recognized phonemes was at least partially mirrored in the mean pupil dilation during the retention interval. Trials leading to three correctly recognized phonemes also led to the lower dilation values in this time-window compared to all other performance levels, while the mean pupil dilation observed when two phonemes were recognized was lower than when no phonemes could be recognized. If patients could recognize three phonemes, they would quickly disengage from the task, and pupil traces go back toward baseline rapidly, leading to lower average dilation values during this period. On the contrary of patients had no (0) or little phonemes recognized (1) the response delay was a period of rehearsing and cognitive effort to try and find an answer and mean pupil dilation remained higher (Figure 6). This effect was very well described by Urai et al. (2017), who showed that the pupil response after choice but before feedback was modulated by performance (incorrect trials leading to larger pupil dilation responses than correct trials) but also by uncertainty regarding response selection. Our results suggest that during the perception of words containing three phonemes, certainty regarding the word to repeat builds up as the number of correctly identified phonemes increases and pupil dilation decreases during the retention interval.

### CI Hearing Experience and Listening Effort

Interestingly, the mean pupil dilation value during the retention interval for correct trials showed a significant correlation with the subjective hearing experience of CI patients as evaluated with the SSQ questionnaire. In particular, the spatial hearing and total SSQ scores showed a significant correlation with mean pupil dilation during the retention interval. Even though these correlations were calculated on relatively small samples (*N* = 10) and should be confirmed with larger groups of CI users, this observation could suggest a general relationship between confidence in own speech perception abilities as reflected in the mean pupil dilation during the retention interval and general subjective hearing abilities. Results from the SSQ questionnaire were otherwise very comparable with what was already observed elsewhere for similar CI patients’ groups (Mertens et al., 2013). Ramakers et al. (2017) have reported a weak but significant correlation between SSQ measures (speech domain) and speech-in-noise tests, but on a larger group of CI patients (*n* = 38). Finally, in a very recent study, a significant correlation was found between the reported daily fatigue and hearing acuity in the form of peak pupil dilation (Wang et al., 2018).

## Conclusion

Altogether, our observations demonstrate that pupillometric measures can be used as an index of listening effort in CI users in a clinical context. From a fundamental level, the pupil dilation measures confirmed previous literature on the peculiar effort needed while noise is added in case of speech recognition task. Moreover, the results demonstrated that pupil response might be indicative of speech recognition performance, in relationship to patients’ subjective experience with speech recognition and hearing in general. While it is noticed that performance with more cognitive cost could be mirrored in pupillometric behavior as a result of increased listening effort in CI users, the interactions between cognitive processes, pupil responses, and speech recognition performance in these patients are complex and should be confirmed and further explored in larger groups of patients. Importantly, further work should investigate the relationship between motivation, age, and peak latency in CI users, or the influence of speech perception motivation and confidence in this population.

## Data Availability Statement

The raw data supporting the conclusions of this article will be made available by the authors, without undue reservation.

## Ethics Statement

The studies involving human participants were reviewed and approved by Comité de Protection des Personnes, Ile de France IV, Paris (ID-RCB N°2017-A00318-45). Written, informed consent was obtained from all included subjects for the publication of any potentially identifiable images or data included in this article.

## Author Contributions

FR, MH, MA, and IM designed the experiment. FR, MA, M-PT, DD, and GL acquired data and managed patients’ appointments and clinical follow-up around the study. MH, CK, and TD analyzed the data. FR, MH, CK, and IM wrote the manuscript. CK, FR, MH, and IM provided critical revisions to the submitted manuscript. All authors contributed to the article and approved the submitted version.

## Conflict of Interest

CK, MH, TD, and MA are employees of Oticon Medical, manufacturer of the cochlear implants used by the patients included in this study and receive salaries as part of their employment. The pupillometry material used in this study was funded by the Oticon Medical Scientific and Clinical Research Group. The remaining authors declare that the research was conducted in the absence of any commercial or financial relationships that could be construed as a potential conflict of interest.

## Footnotes

1 http://www.has-sante.fr

2 https://www.scipy.org/
